# Rethinking Ayurvedic antibacterials: a mechanistic case for multi-target phytochemicals as adjuvants against antimicrobial resistance

**DOI:** 10.3389/fphar.2026.1946531

**Published:** 2026-10-22

**Authors:** Hari Narayanan KN, Shanker Shyam Sundhar Panchapakesan

**Affiliations:** Nucleic Acid Engineering Research and Development in SASTRA (NERDS), SASTRA Deemed University, Thanjavur, Tamil Nadu, India

**Keywords:** antibiotic adjuvants, antimicrobial resistance, Ayurveda, multi-target mechanism, phytochemicals, polypharmacology

## Abstract

Antimicrobial resistance (AMR) is now among the foremost threats to global health, and the pipeline of new antibiotics has not kept pace with it. A structural driver of resistance is often overlooked: the dominant “single molecule–single target” paradigm of antibiotic design imposes a well-defined selective pressure that bacteria can escape through a single mutation or acquired gene. This review reframes the antibacterial potential of Ayurvedic herbs around that problem. Rather than cataloguing herbs as candidate “natural antibiotics,” we argue that their value lies in polypharmacology—the simultaneous action of structurally diverse secondary metabolites (flavonoids, tannins, saponins, phenolic acids and terpenoids) on multiple conserved bacterial targets, a combination against which stepwise resistance is evolutionarily improbable. We synthesise the mechanistic evidence for representative Ayurvedic plants including *Calotropis procera*, *Azadirachta indica*, *Ocimum sanctum*, the *Acacia* genus and *Asparagus racemosus*, organising it by bacterial target rather than by species: cell-wall biosynthesis, membrane integrity, and essential enzymes such as DNA gyrase and β-lactamase. We then examine two roles in which multi-target phytochemicals address failures of conventional therapy: the disruption of biofilms and quorum sensing, and synergy with existing antibiotics through efflux inhibition, β-lactamase suppression and extracellular-matrix degradation. Finally, we assess the translational barriers: reproducibility, heavy-metal contamination and the scarcity of clinical trials that currently confine most of this evidence to *in vitro* studies. We conclude that the most credible near-term contribution of Ayurvedic pharmacology to the AMR crisis is not as a replacement for antibiotics but as a source of multi-target adjuvants that restore and extend their efficacy.

## 1 Introduction

Antimicrobial resistance (AMR) has become one of the central challenges facing the global healthcare system. Despite numerous national and international action plans, global resistance rates have not fallen appreciably; the overuse and misuse of antibiotics in both medicine and agriculture remain principal drivers (Dadgostar, 2022). Resistance also spreads horizontally: mobile genetic elements allow resistant genes to move between related and unrelated bacteria, so that resistance arising in one species rapidly seeds resistance across many others (Dadgostar, 2022). The consequences are economic as well as clinical. The Organisation for Economic Co-operation and Development projects that AMR could cause roughly 2.4 million deaths across Europe, North America and Australia over 3 decades, at a cost of up to US$3.5 billion per year (Hofer, 2019), while the US CDC estimates that around two million people are affected worldwide. India carries a disproportionate burden - an AMR-associated mortality of approximately 416.75 per 100,000, close to twice the US figure, with methicillin-resistant *Staphylococcus aureus* accounting for about 41% of *S. aureus* isolates (Patwardhan and Payyappalli, 2021).

Efforts to counter AMR have concentrated overwhelmingly on discovering new molecules that inhibit a single bacterial target with high specificity. That precision is also a liability. A single, well-defined target defines a single, well-defined route of escape: one mutation in the target, one acquired efflux pump, or one hydrolytic enzyme can abolish a drug’s activity, and the strong selective pressure exerted by a potent single-target agent actively favours such variants (Tenover, 2006; Holmes et al., 2016). Viewed this way, resistance is not merely a consequence of misuse but a predictable outcome of the design paradigm itself - a point that motivates the search for antibacterial strategies built on a different logic.

Ayurveda, India’s traditional system of medicine, embodies precisely such a logic. It does not typically seek to eliminate a pathogen with one therapeutic molecule; instead, it uses complex, multi-component plant preparations intended to restore the balance between host and environment so that the body clears the infection (Ramaswamy, 2018; Mishra et al., 2001). For much of the modern era this holistic framing was dismissed as unquantifiable and incompatible with reductionist science. Contemporary pharmacology, however, has independently arrived at a concept that gives it a mechanistic footing—polypharmacology, the deliberate use of agents that act on several targets at once (Nasim et al., 2022). Ayurvedic antibacterial preparations are, in effect, naturally occurring polypharmacological mixtures.

This review is organised around that reframing. Rather than presenting Ayurvedic plants as candidate stand-alone antibiotics, a framing common in the existing literature and one that adds little to it, we treat them as a source of multi-target chemistry and ask three questions. First, how do their secondary metabolites act, in molecular terms, on conserved bacterial structures, and why does engaging many of them at once make resistance improbable? Second, can they dismantle biofilms, a bacterial lifestyle against which conventional antibiotics are largely ineffective? Third, can they be combined with existing antibiotics to restore activity that resistance has eroded? Table 1 lists the plants evaluated here against the WHO/DBT Indian Priority Pathogen List (WHO and DBT, 2021); because the phytochemistry of every candidate cannot be surveyed exhaustively, the most frequently cited plants (Figure 1), the plant parts used (Figure 2) and their principal active constituents are examined in detail.

**TABLE 1:** Plants evaluated as antimicrobials against bacteria showing AMR.

Pathogen		Medicinal plants	Author
Enterobacteriaceae	*Klebsiella pneumoniae*	*Azadirachta indica* (leaves)	Rathod et al. (2012)
		*Azadirachta indica* (bark)	
		*Ocimum sanctum* (leaves)	
		*Pinus roxburghii* (stem bark)	Sharma et al. (2016)
		*Pinus wallichiana* (stem bark)	
		*Azadirachta indica* (leaves)	Faujdar et al. (2020)
		*Calotropis procera* (twigs)	Parabia et al. (2008)
		*Calotropis procera* (latex)	
		*Acacia arabica* (leaf)	Adhikari and Rangra (2023)
	*Escherichia coli*	*Azadirachta indica* (leaves)	Rathod et al. (2012)
		*Azadirachta indica* (bark)	
		*Ocimum sanctum* (leaves)	
		*Ocimum sanctum* (leaves), oil from fresh leaves	Mondal et al. (2007)
		*Ocimum sanctum* (leaves), oil from dried leaves	
		*Boerhavia diffusa* (leaves)	Rema shree and Remashree (2014)
		*Asparagus racemosus* (root)	Jadhav (2018)
		*Azadirachta indica* (leaves)	Faujdar et al. (2020)
		*Calotropis procera* (twigs)	Parabia et al. (2008)
		*Calotropis procera* (latex)	
		*Acacia arabica* (leaf)	Adhikari and Rangra (2023)
		*Acacia rigidula* (leaf)	
		*Acacia polyacantha* (bark)	
		*Acacia caesia* (stem)	
		*Acacia auriculiformis* (phyllode)	
		*Astragalus membranaceus* (root)	Balachandar et al. (2012)
Non-fermenting bacteria	*Acinetobacter baumannii*	*Semecarpus anacardium* (seed)	Purushothaman et al. (2017)
		*Azadirachta indica* (leaves)	Faujdar et al. (2020)
	*Pseudomonas aeruginosa*	*Asparagus racemosus* (root)	Jadhav (2018)
		*Pinus wallichiana* (stem bark)	Sharma et al. (2016)
		*Pinus gerardiana* (stem bark)	
		*Plumeria obtusa* (leaves)	Singh and Verma (2019)
		*Azadirachta indica* (leaves)	Faujdar et al. (2020)
		*Calotropis procera* (twigs)	Parabia et al. (2008)
		*Calotropis procera* (latex)	
		*Acacia arabica* (leaf)	Adhikari and Rangra (2023)
		*Acacia rigidula* (leaf)	
		*Acacia mearnsii* (leaf)	
​	*Staphylococcus aureus*	*Azadirachta indica* (leaves)	Rathod et al. (2012)
		*Azadirachta indica* (bark)	
		*Ocimum sanctum* (leaves)	
		*Asparagus racemosus* (root)	Jadhav (2018)
		*Pinus roxburghii* (stem bark)	Sharma et al. (2016)
		*Pinus wallichiana* (stem bark)	
		*Plumeria obtusa* (leaves)	Singh and Verma (2019)
		*Calotropis procera* (twigs)	Parabia et al. (2008)
		*Calotropis procera* (latex)	
		*Acacia arabica* (leaf)	Adhikari and Rangra (2023)
		*Acacia rigidula* (leaf)	
		*Acacia auriculiformis* (phyllode)	
		*Acacia karoo* (roots)	
		*Acacia polyacantha* (bark)	
		*Acacia caesia* (stem)	
	*Enterococcus species*	*Boerhavia diffusa* (leaves)	Rema shree and Remashree (2014)
		*Acacia rigidula* (leaf)	Adhikari and Rangra (2023)
​	*Salmonella species*	*Nyctanthes arbortristis* (flower)	Pasha et al. (2009)
		*Garcinia indica* (leaf)	
		*Rubia cordifolia* (root)	
		*Azadiracta indica* (seed, leaf)	
		*Momordica charantia* (seed)	
		*Ocimum sanctum* (leaves), oil from fresh leaves	Mondal et al. (2007)
		*Ocimum sanctum* (leaves), oil from dried leaves	
		*Plumeria obtusa* (leaves)	Singh and Verma (2019)
		*Calotropis procera* (twigs)	Parabia et al. (2008)
		*Calotropis procera* (latex)	
		*Acacia arabica* (leaf)	Adhikari and Rangra (2023)
		*Acacia nilotica* (pods)	
		*Astragalus membranaceus* (root)	Balachandar et al. (2012)
	*Streptococcus pneumoniae*	*Asparagus racemosus* (root)	Jadhav (2018)
	*staphylococcus*, coagulase-negative	*Asparagus racemosus* (root)	Jadhav (2018)
		*Calotropis procera* (twigs)	Parabia et al. (2008)
		*Calotropis procera* (latex)	
		*Acacia arabica* (leaf)	Adhikari and Rangra (2023)
		*Astragalus membranaceus* (root)	Balachandar et al. (2012)
​	*Shigella* sp	*Ocimum sanctum* (leaves), oil from fresh leaves	Mondal et al. (2007)
		*Ocimum sanctum* (leaves), oil from dried leaves	
	*Haemophilus influenzae*	*Sida rhombifolia* (aerial parts)	Rai et al. (2017)
	*Enterobacter* spp.*,*	*Azadirachta indica* (leaves)	Faujdar et al. (2020)
		*Calotropis procera* (twigs)	Parabia et al. (2008)
		*Calotropis procera* (latex)	
		*Acacia arabica* (leaf)	Adhikari and Rangra (2023)
	*Serratia* spp.*,*	*Calotropis procera* (twigs)	Parabia et al. (2008)
		*Calotropis procera* (latex)	
		*Boerhavia diffusa* (leaves)	Rema shree and Remashree (2014)
		*Azadirachta indica* (leaves)	Faujdar et al. (2020)
		*Acacia karoo (leaves and roots)*	Adhikari and Rangra (2023)
	*Providencia* spp.*,*	*Acacia rigidula* (leaf)	
	*Helicobacter pylori*	*Bacopa monniera* (bacoside A)	Goel et al. (2003)
	*Campylobacter*	*Astragalus membranaceus* (root)	Balachandar et al. (2012)

**FIGURE 1:**
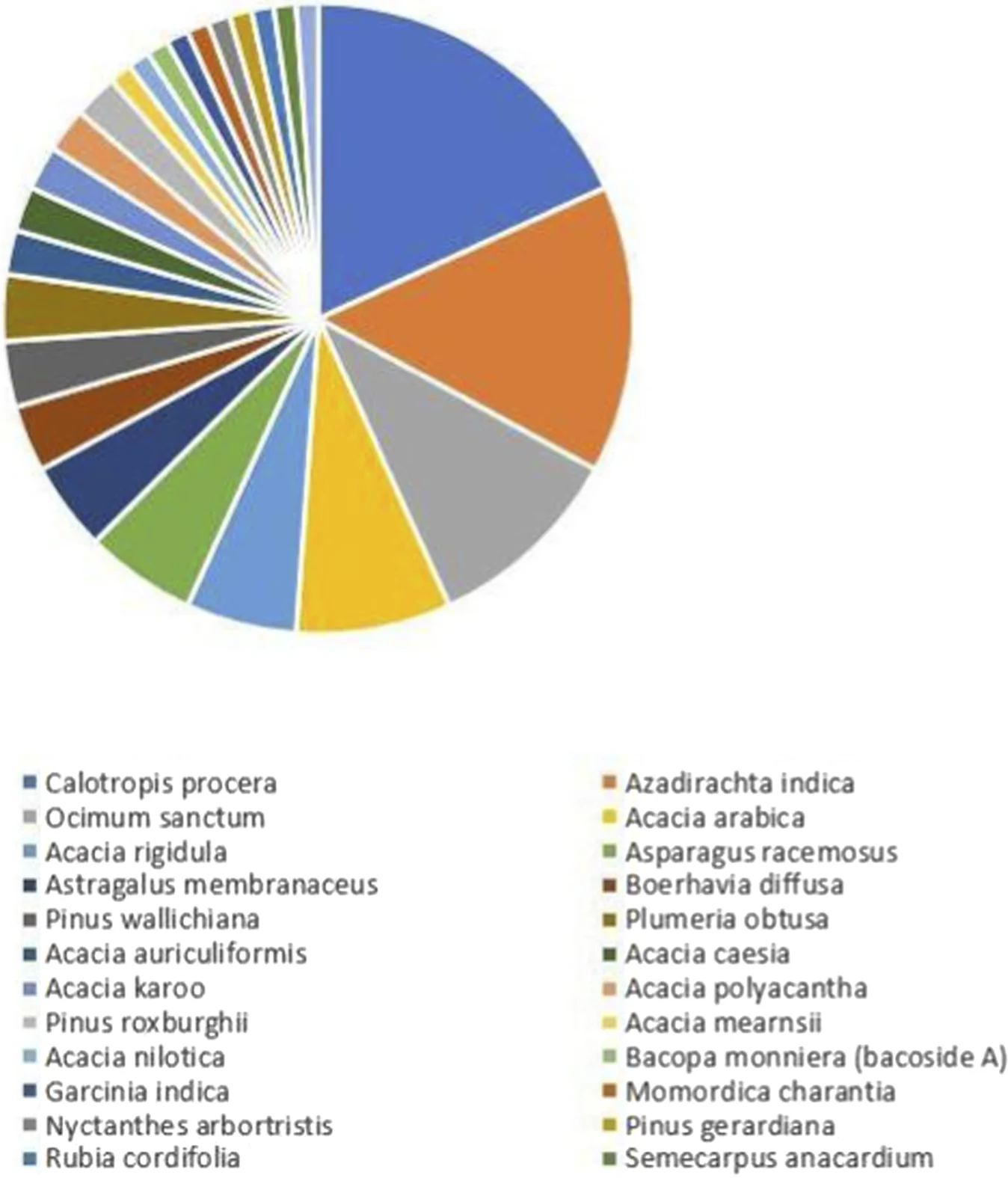
Frequency by which the plants mentioned have been used against pathogenic bacteria.

**FIGURE 2:**
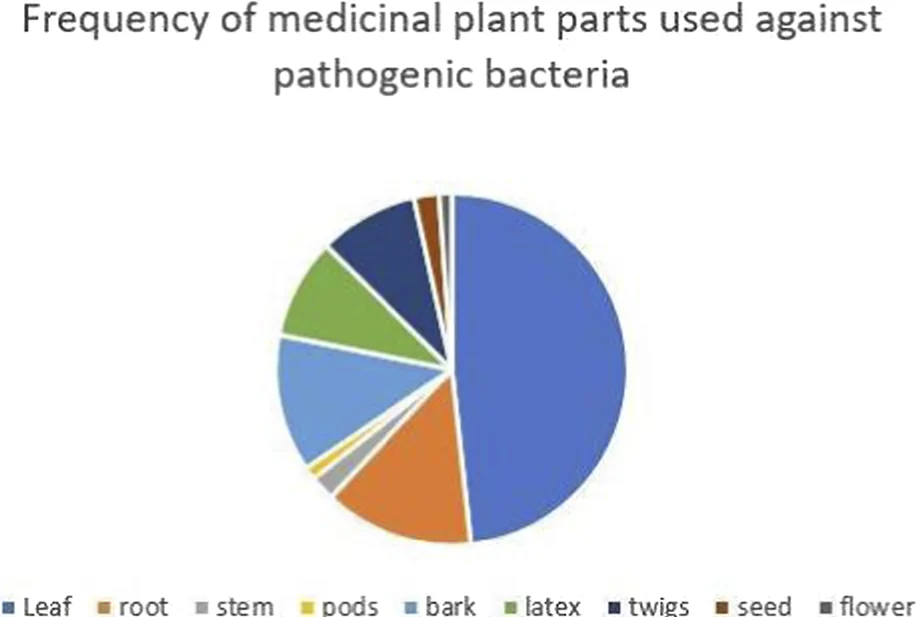
Frequency of medicinal plant parts used against pathogenic bacteria.

## 2 Methodology

A comprehensive literature review was conducted to evaluate the antibacterial potential of Ayurvedic herbs and their polypharmacological effects against bacteria identified as priority pathogens by the WHO. The review focused on identifying evidence for the mechanistic action of phytochemicals against resistance, rather than cataloguing plants as mere stand-alone antibacterials.

### 2.1 Database search strategy

Peer-reviewed articles were sourced from databases including Google Scholar, PubMed and Scopus. Primary search terms included “antimicrobial resistance,” “Ayurveda,” “polypharmacology,” “antibiotic adjuvants,” “phytochemicals” and “multi-target mechanism.” Further searches were conducted using the traditional plant names *Calotropis procera*, *Azadirachta indica*, *Ocimum sanctum*, the *Acacia* genus, and *Asparagus racemosus*. These five genera were selected as the focal subjects of this review because they were both the most frequently cited antibacterial plants in the literature surveyed (Figure 1) and had the most extensively characterised phytochemical mechanisms of action; this was a qualitative judgement based on citation frequency and depth of mechanistic reporting, rather than a numeric inclusion threshold, and the remaining plants identified in the search are listed in Table 1 without detailed mechanistic treatment.

### 2.2 Inclusion and exclusion criteria

Studies were included if they provided mechanistic evidence for the site-specific actions of phytochemicals such as flavonoids and tannins against bacteria on the WHO Indian Pathogen Priority List; studies demonstrating activity against biofilm formation, quorum sensing, or synergy with antibiotics were prioritised. Studies were excluded if they reported plants merely as stand-alone antibacterials without detailing the underlying mechanism of action of their phytochemicals against bacterial targets, together with non-peer-reviewed articles and studies focused on non-human pathogens.

### 2.3 Scope and nature of the evidence

This review draws exclusively on previously published, peer-reviewed literature; it reports no new experimental, *in-vitro*, *in silico* or clinical data of the authors’ own. All schematic figures (Figures 1–3) are original illustrations prepared by the authors to synthesise and organise findings already reported in the cited sources, and none depicts unpublished or original experimental results. As detailed in Section 8, the underlying evidence base for the multi-target mechanisms discussed here is drawn overwhelmingly from *in-vitro* and *in silico* studies, with clinical validation in humans still scarce; conclusions about mechanism and translational potential should be read with this preclinical status in mind.

**FIGURE 3:**
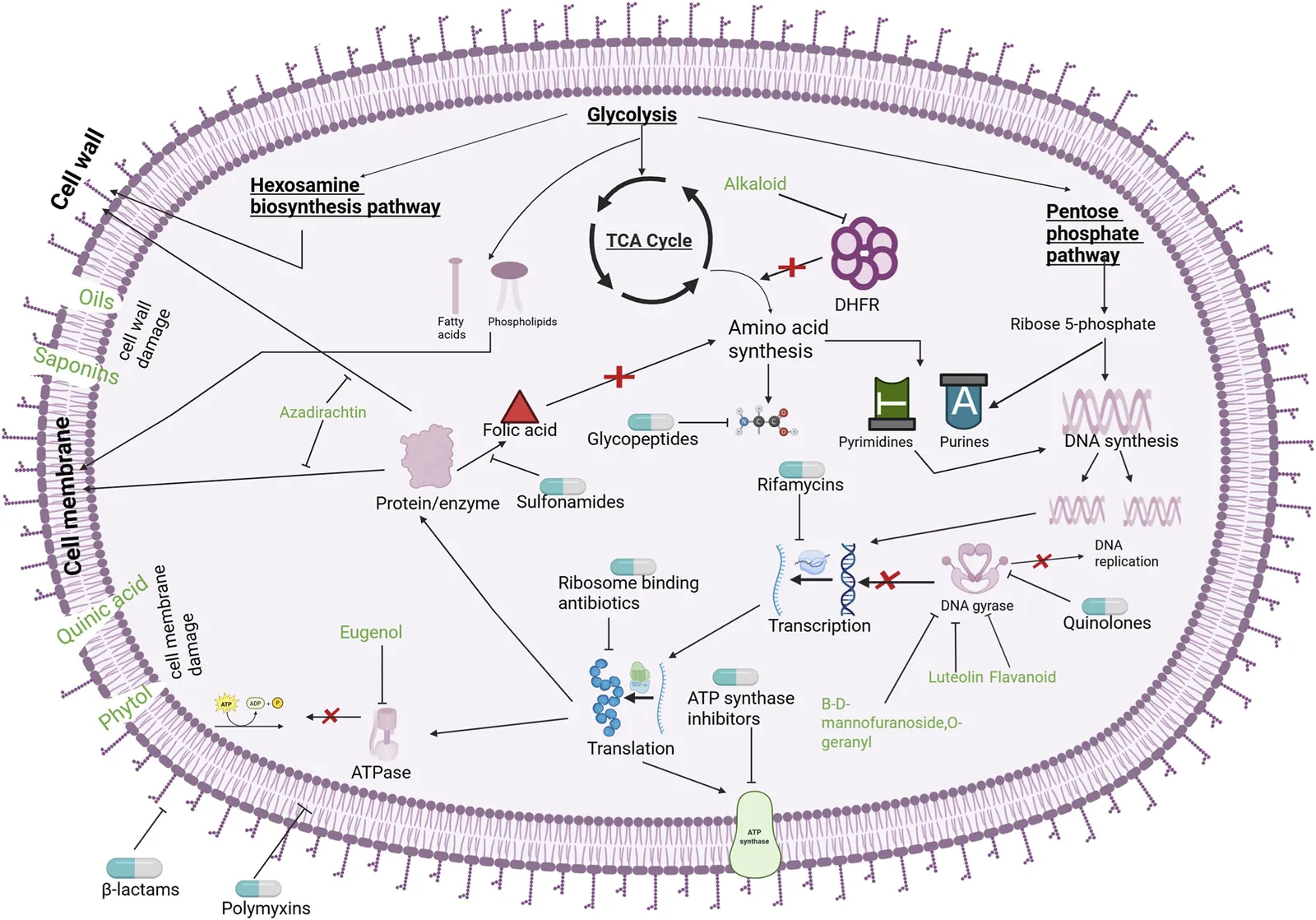
The effects of plant bioactive molecules are compared with different classes of antibiotics. The plant molecules are shown in green text, and the antibiotics are shown as capsules. While conventional antibiotics exert focused selective pressure that predictably drives resistance, phytochemical classes such as flavonoids, tannins, saponins, phenolic acids, and terpenoids simultaneously engage multiple conserved bacterial targets. The diagram maps these synergistic interactions, detailing cell wall and membrane disruption (e.g., peptidoglycan permeabilization and lipid bilayer disordering), the suppression of essential metabolic and replicative enzymes (e.g., DNA gyrase and dihydrofolate reductase), and the direct neutralization of resistance machinery, such as efflux pumps and β-lactamases. By illustrating this coordinated polypharmacology, the figure highlights how phytochemical mixtures bypass established single-target resistance pathways and render stepwise evolutionary adaptation highly improbable. This is a schematic synthesis of mechanisms reported in the peer-reviewed sources cited in Sections 4, 5; it does not depict unpublished, original or unvalidated experimental data, and the underlying evidence for each interaction is predominantly *in-vitro* or *in silico* rather than clinical (see Section 8).

## 3 The mechanistic basis of resistance: why single targets fail

Antimicrobial resistance is, at its simplest, the capacity of bacteria to survive drug concentrations that would previously have inhibited them - in molecular terms, a heritable upward shift in the minimum inhibitory concentration that can then be passed to neighbouring cells and drive the emergence of new resistant variants (Acar, 2015). The phenomenon is not new. Penicillin-resistant *S. aureus* emerged in hospitals within a decade of the drug’s introduction, producing β-lactamase to hydrolyse penicillin, and resistance to structurally unrelated antibiotics soon followed (WHO, 1983). Alexander Fleming anticipated this in his 1945 Nobel lecture, warning that sub-lethal, unsupervised exposure would inevitably breed resistant organisms (Sullivan, 2018).

Modern antibiotics act through a small number of mechanisms: inhibition of cell-wall synthesis (β-lactams, glycopeptides), of protein synthesis (macrolides, tetracyclines), of nucleic-acid synthesis (fluoroquinolones, rifampin), and of folate metabolism (trimethoprim–sulfamethoxazole), together with disruption of the cell membrane (polymyxins, daptomycin) (Tenover, 2006). Against each, bacteria deploy a corresponding countermeasure - modification of the drug target, increased efflux, enzymatic inactivation of the drug, or protection of the target by dedicated DNA-binding proteins (Holmes et al., 2016). The decisive point for what follows is that every one of these escape routes neutralises an agent that depends on a *single* target. A therapy that instead engages several essential targets simultaneously cannot be defeated by any one of these adaptations, the conceptual basis for the phytochemical strategies examined in the remainder of this review.

## 4 Polypharmacology: why multi-target mixtures resist resistance

Across the plants surveyed here, a consistent pattern emerges. Their antibacterial activity rests not on plant-specific mechanisms so much as on a shared repertoire of secondary-metabolite classes principally flavonoids, tannins, saponins, phenolic acids and terpenoids that recur across taxonomically distant species. These compounds are so widespread precisely because they target features common to most bacteria: the cell wall, the cell membrane, and conserved enzymes such as DNA gyrase (a convergence summarised in (Figure 3).

This convergence is the source of their value against resistance. Where a single-target antibiotic imposes one selective pressure that one mutation can relieve, a phytochemical mixture presses on several essential systems at once. For a bacterium to escape, it would need to acquire multiple, simultaneous adaptations, an event whose probability is the product of several already-rare mutation frequencies, and therefore markedly less likely than escape from a single-target agent. Direct experimental support for this comes from serial-passage resistance-evolution assays on a multi-component historical antimicrobial, which found that *S. aureus*, *A. baumannii* and *P. aeruginosa* found it markedly harder to evolve resistance against the multi-target mixture than against single-target controls (Orababa et al., 2026). The observation that traditional remedies act through the synergy of many constituents, rather than one dominant molecule, is consistent with this account (Nasim et al., 2022). This is the essence of polypharmacology: the coordinated action of chemically diverse molecules on multiple targets to improve host physiology (Nasim et al., 2022). The sections that follow set out the molecular evidence for this multi-target action, organised by the bacterial structure or process each class of compound attacks rather than by plant, an arrangement that makes the multi-target logic explicit.

Bacteria also employ complex transcriptional and epigenetic modifications to induce dormancy when threatened by antibiotics, a state distinct from classical resistance in that these dormant cells can survive even multidrug attack. One route by which bacteria clear multiple drugs from the cell is active efflux, as in the persister cells of *E. coli*, which rely on pumps such as *tolC* (Van den Bergh et al., 2017). Conventional antibiotics rest on a single-molecule, single-target system that bacteria can defeat through a single point mutation; phytochemicals such as polyphenols instead act by polypharmacology, attacking multiple bacterial targets at once - cell-wall and membrane disruption, inhibition of key enzymes, and disruption of metabolic and signalling pathways, a multifront attack that reduces the likelihood of resistance emerging (Bayode et al., 2026).

Essential oils illustrate this multitarget attack starting at the cell envelope: rich in polyphenols and terpenoids, whose lipophilicity gives them high affinity for the proteins and glycoproteins of the cell membrane, they increase membrane permeability and cause leakage of cellular contents that compromises cell integrity (Bayode et al., 2026). Bacteria exposed to antimicrobials that target the cell envelope mount stress responses that remodel their lipidome and membrane proteome in defence altering the length, branching or saturation of fatty-acyl chains, changing lipid composition, or synthesising membrane-protective proteins (Willdigg and Helmann, 2021) and can further form biofilms that use chemical gradients to lower outer-membrane permeability, impeding antibacterial entry (Uruén et al., 2021).

## 5 Mechanistic evidence across conserved bacterial targets

The plants examined below are among the most frequently cited antibacterials in Ayurvedic practice. *Calotropis procera*, described in the *Charaka Samhita* and *Sushruta Samhita* for wound healing, contains glycosides, flavonoids, triterpenes, alkaloids, steroids, saponins, proteins and enzymes (Amini et al., 2021; Yadav and Kumar, 2022). *Azadirachta indica* (neem) is a mainstay of traditional therapeutics, its activity distributed across azadirachtin, nimbin, salannin and several flavonoids (Mandal and Mandal, 2011). *Ocimum sanctum* (Tulsi), its Sanskrit name meaning “incomparable” is valued for an unusually broad medicinal range (Bindhani and Panigrahi, 2015). The *Acacia* genus (Mimosaceae) supplies wound-healing, antiseptic and antidiabetic remedies across several species (Adhikari and Rangra, 2023; Batiha et al., 2022), and *A. racemosus* (Shatavari) is used as an antioxidant, antimicrobial and hepatoprotective tonic (Selvaraj et al., 2019). Grouping their constituents by bacterial target makes the multi-target argument concrete; the mechanisms are summarised in Figure 3.

### 5.1 Cell wall and penicillin-binding proteins

The bacterial cell wall is a frequent point of attack. Flavonoids illustrate the mechanism: quercetin, an abundant flavonoid in *A. indica*, interacts with the peptidoglycan layer to increase permeability and damage the wall, an effect reflected in its stronger activity against Gram-positive than Gram-negative organisms as was evident from *in-vitro* validation (Wang et al., 2018). *In-vitro* validation demonstrates that tannins inhibit cell-wall synthesis by forming irreversible complexes with the proline-rich proteins of the wall (Gupta and Kaur, 2021) and, more broadly, inactivate bacterial adhesins, enzymes and envelope transport proteins while altering cell morphology (Agarwal et al., 2010). Several *A. indica* constituents like azadirachtin, salannin, meliantriol, mahmoodin and nimbin similarly interfere with cell-wall and membrane synthesis *in-vitro* (Mandal and Mandal, 2011). Combined *in silico* and *in-vitro* studies provide direct evidence for wall targeting *via* the *A. indica* compound β-D-mannofuranoside, O-geranyl, which binds with high affinity to the penicillin-binding proteins PBP2X and PBP4, as well as to tyrosyl-tRNA synthetase and DNA gyrase (Altayb et al., 2022). In the *Acacia* genus, acetyl eugenol from *Acacia nilotica* binds the protein kinases *Mt*PknE and *Mt*PknB of *Mycobacterium tuberculosis in-vitro*, inhibiting the cell growth and division these kinases regulate (Abdulhamid et al., 2021).

### 5.2 Membrane integrity

A second, recurrent target is the cytoplasmic membrane. *In-vitro* models indicates that in *C. procera*, flavonoids bind both cell-wall and membrane components to disrupt membrane integrity, and additionally inhibit bacterial enzymes by engaging their active sites through hydroxyl groups (Amini et al., 2021). Eugenol, the principal antibacterial of *O. sanctum*, alters membrane permeability *in-vitro*, so that intracellular contents leak and the cell dies, while also inhibiting membrane ATPase. Saponins, which carry sugar moieties, insert into the lipid bilayer and disorder the acyl chains of the membrane lipids; the disruption scales with the number of sugar units, ultimately raising permeability to the point of lysis during *in-vitro* assays (Ondevilla et al., 2023). Phenolic acids act through their amphipathic character: *In-vitro* validation shows that in *A. racemosus* the hydrophilic portion engages the polar membrane surface while the hydrophobic portion buries within the bilayer, and the molecules further inactivate proteins by binding their sulfhydryl groups (Viuda-Martos et al., 2010; Zhang et al., 2019). Gallic acid, a major constituent of *A. arabica*, owes its membrane-rupturing toxicity to three hydroxyl groups, causing leakage of cell contents, pH change and chelation of divalent cations (Farag et al., 2015; Wianowska and Olszowy-Tomczyk, 2023); the related methyl gallate of *A. farnesiana* compromises membrane integrity, lowers cytoplasmic pH and hyperpolarises the membrane until treated cells can no longer generate ATP (Batiha et al., 2022). Comparable membrane-directed *in-vitro* activity is reported for the diterpene phytol of *Sida rhombifolia*, which induces potassium-ion loss from *S. aureus* (Inoue et al., 2005), for the oils of *Semecarpus anacardium*, which damage the wall and membrane and deplete the proton-motive force (Bagewadi et al., 2012), and for quinic acid from *Pinus* species (Ramos et al., 2022).

### 5.3 Essential enzymes and nucleic-acid synthesis

A third group of constituents inhibits enzymes indispensable to replication and metabolism. DNA gyrase is a common target: *in-vitro* studies show that azadirachtin and quercetin inhibit both gyrase and ATPase activity (Wang et al., 2018; Khare and Kamble, 2025), and *in silico* molecular-docking studies attribute the potent anti-*S. aureus* activity of luteolin, a flavone from *Bacopa monnieri*, to its high affinity for the gyrase binding site (Emran et al., 2015). Phenolic compounds inhibit dihydrofolate reductase which is required for the supercoiling activity of gyrase and alter the ATP-binding site of gyrase B while promoting topoisomerase-IV-mediated DNA cleavage *in-vitro*, together suppressing replication (Akinduti et al., 2024). Alkaloids, such as those in *Momordica charantia* leaves, likewise inhibit nucleic-acid synthesis and dihydrofolate reductase, while the flavonoids, tannins and saponins that accompany them impair cell-wall activity, energy metabolism and protein retention respectively *in-vitro* (Masithoh et al., 2019). Beyond replication, some constituents strike at metabolism: asparacemosones, a spirosteroid from *A. racemosus*, inhibits α-glucosidase and thereby the organism’s carbohydrate metabolism (Tantapakul et al., 2020), and catechin and proanthocyanidins from *Pinus* species inhibit growth by hydrogen-bonding and hydrophobic interaction with bacterial enzymes (Ramos et al., 2022).

### 5.4 Disarming the resistance machinery directly

A final and strategically important group targets the resistance machinery itself. Rutin (quercetin-3-O-rutinoside), a flavonoid of *C. procera*, inhibits β-lactamases, efflux pumps and biofilm formation in both *in silico* and *in-vitro* studies, and significantly downregulates around fifteen biofilm-formation genes in *Klebsiella pneumoniae.* Rutin hydrate showed MICs in the range of 128–1,024 μg/mL against various tested strains and a FIC index of less than 0.5 against 4 strains when combined with amikacin, denoting synergy (Nenaah, 2013; Miklasińska-Majdanik et al., 2023) (Table 2). Quercetin itself can restore drug susceptibility: in amoxicillin-resistant *Staphylococcus epidermidis* it inhibits β-lactamase and re-sensitises the organism to the antibiotic *in-vitro* with a Fractional Inhibitory Concentration (FIC) index of 0.5. (Siriwong et al., 2016) (Table 2). Soluble laticifer proteins from *C. procera*, proteolytic in character (proteases, protease inhibitors or peptides), add a further, protein-based mode of antibacterial action *in-vitro* (Saher et al., 2023). This capacity to neutralise the very enzymes that confer resistance foreshadows the adjuvant role developed in Section 7.

**TABLE 2:** Consolidated quantitative synergy and adjuvant evidence for the phytochemical mixtures reviewed.

Compound	Plant source	Target organism(s)	Concentration/mic	Effect	FIC index	Source
Rutin hydrate	*C*. *procera*	Various Gram-negative/positive strains	128–1,024 μg/mL	Antibacterial; downregulates ∼15 biofilm-formation genes in *K. pneumoniae*	<0.5 (with amikacin, 4 strains)	Nenaah (2013), Miklasińska-Majdanik et al. (2023)
Quercetin	*A. indica*	*Staphylococcus epidermidis* (amoxicillin-resistant)	Not specified (β-lactamase inhibition assay)	Restores amoxicillin susceptibility	0.5	Siriwong et al. (2016)
Quercetin	*A. indica*	*S. epidermidis*	125–1,000 μg/mL	Reduces biofilm formation (ica downregulation)	—	Mu et al. (2021)
Catechin	*A. indica*	*Porphyromonas gingivalis*	Not specified	Suppresses quorum sensing by 94.56% ± 2.56% (LuxS reduction); degrades EPS	—	Lahiri et al. (2021)

## 6 Dismantling biofilms: a target conventional antibiotics miss

Biofilms, multicellular bacterial aggregates encased in a self-produced extracellular matrix, underlie many persistent infections and are notoriously refractory to conventional antibiotics, making them a priority target for new agents (Kadam et al., 2020). Their resistance is multifactorial. The matrix itself shields resident cells from antibiotics; horizontal gene transfer proceeds far more efficiently within biofilms than among planktonic cells, spreading resistance genes to susceptible neighbours; and metabolically dormant “persister” cells buried in the matrix survive drugs that require active targets, seeding chronic, relapsing infection. Biofilms are typically polymicrobial, and the close packing of cells intensifies the interspecies interactions and quorum-sensing signalling that elevate both virulence and resistance, while biofilm-associated efflux keeps intracellular antibiotic concentrations sub-lethal - itself a route to further resistance (Liu et al., 2024).

Multi-target plant chemistry is well suited to this problem, and much of its anti-biofilm action works by interrupting the same processes (Kadam et al., 2020). Quorum sensing is a frequent point of intervention: docking studies show that cardenolides such as strophanthidin bind MurG, a glycosyltransferase required for biofilm formation (Rodríguez-Macías et al., 2025; Zhang et al., 2021). Catechin from *A. indica* degrades the extracellular polymeric substances (EPS) that hold biofilms together and suppresses quorum sensing by 94.56% ± 2.56% in organisms such as *Porphyromonas gingivalis* by reducing LuxS activity (Lahiri et al., 2021) (Table 2). Because surface attachment is the first step in biofilm formation (Armbruster and Parsek, 2018), the ability of tannins to inactivate bacterial adhesins blocks biofilms at their inception (Agarwal et al., 2010); tannin-rich fractions of *Terminalia catappa* and *T. chebula* further inhibit quorum sensing in *Pseudomonas aeruginosa*, and tannic acid induces transglucosylase activity in *S. aureus* (Armbruster and Parsek, 2018).

## 7 Restoring antibiotic efficacy: phytochemical–antibiotic synergy

The biofilm lifestyle also explains much antibiotic failure, and it points to the most immediately translatable role for these compounds. Within a biofilm, bacteria resist antibiotics through drug inactivation or modification, target alteration, efflux and altered metabolic pathways (Maurice et al., 2018), and antibiotics frequently cannot penetrate the EPS to reach their target at all (Bonincontro et al., 2023). Phytochemicals can remove these obstacles - preventing biofilm formation, degrading the extracellular matrix, and inhibiting essential proteins, DNA replication and efflux pumps, so that co-administered antibiotics regain access and potency (Kungwani et al., 2024). Several are already documented as synergistic. Quercetin at concentrations ranging from 125–1,000 μg/mL significantly reduced biofilm formation in *S. epidermidis.* It reduces the adhesion of biofilm-forming *S. epidermidis* by downregulating the intercellular-adhesion locus (ica) and lowering polysaccharide-intercellular-adhesin production (Mu et al., 2021) (Table 2), and, as noted above, suppresses β-lactamase to restore amoxicillin activity (Siriwong et al., 2016); catechin degrades biofilm EPS to admit antibiotics to their targets (Lahiri et al., 2021). This adjuvant role, restoring the efficacy of drugs that resistance has compromised, rather than replacing them, is, we argue, the most realistic near-term clinical contribution of Ayurvedic phytochemistry to the AMR problem.

Realising this adjuvant role, however, depends on demonstrating true synergy rather than mere additivity, and no standard test currently exists for antimicrobial interactions. Commonly used assays such as time-kill methods each carry their own advantages and limitations and can over- or under-estimate an interaction, at times misclassifying an additive effect as synergistic, a problem that motivates the use of rigorous mathematical models of antimicrobial interaction (Hakeem et al., 2019). Researchers should therefore employ models such as Loewe additivity, based on zero-interaction theory; the Fractional Inhibitory Concentration (FIC) index applies this principle directly to *in-vitro* checkerboard assays under a strict, standardised numerical threshold, with an FIC index of 1 indicating additivity and values below or above 1 indicating synergy or antagonism, respectively (Meletiadis et al., 2010). Comparing antimicrobial interactions against such mathematical benchmarks reduces subjective interpretation, and adopting this quantitative, cutoff-based approach helps confirm that an enhanced effect from combining phytochemicals with antibiotics reflects true synergy rather than simple additivity, and that the combination is translationally reliable (Hakeem et al., 2019; Meletiadis et al., 2010).

## 8 Translational barriers

The evidence above is mechanistically compelling but largely pre-clinical, and three obstacles stand between it and the clinic. The first is reproducibility. Antibacterial activity varies between batches of the same species grown under different seasons, harvest times and geographies, and the sheer diversity of species and phytochemical profiles makes potency difficult to standardise (Masota et al., 2021; Busia, 2024). Analytical standardisation by techniques such as HPLC-ESI-MS/MS is feasible but slow and costly (Jarouche et al., 2019). The second is safety: heavy-metal contamination is a documented hazard, with some commercial preparations carrying lead, mercury or arsenic at 100–10,000 times recommended limits (Jarouche et al., 2019), and a 2010–2019 US investigation found markedly elevated heavy-metal exposure among users of Rasa Shastra preparations, which intentionally incorporate processed metals (Hore et al., 2022). Adherence to Good Manufacturing Practice and modern quality control can mitigate this and would improve global acceptance (Gupta, 2024). The third is the clinical-trial gap. Batch variability, inconsistent manufacturing and limited safety and efficacy data have kept most validation *in-vitro* (Parveen et al., 2015), and few academic studies proceed to isolate a defined active principle or to pursue the lengthy, capital-intensive path of drug development (Abdallah et al., 2023). These constraints are the principal reason herbal antibacterials cannot yet serve as a first-line response to resistant infection and the reason the adjuvant and standardised-extract routes, which tolerate mixture complexity better than single-molecule development, are the more pragmatic near-term goals. Taken together, these obstacles mean that, at present, the evidence base for the multi-target mechanisms described in this review is overwhelmingly preclinical, and nothing here should be read as establishing clinical readiness. The three subsections below examine the remaining barriers in more depth: Section 8.1 reviews what clinical evidence and human-safety data do exist despite the trial gap, Section 8.2 turns to a further, pharmacokinetic barrier - the poor bioavailability and stability that limit even safe, well-studied phytochemicals from reaching their bacterial targets and Section 8.3 addresses the regulatory complexity that multicomponent, polypharmacological preparations face on the path to approval.

### 8.1 Clinical evidence and human safety

Although most phytomedicine research remains pre-clinical, a limited body of clinical research and a handful of established formulations do provide evidence of efficacy and human safety. Despite promising *in-vitro* results, most phytodrugs remain ineligible for human trials because of toxicity and poor bioavailability (Abdallah et al., 2023). Some, however, have established clinical efficacy: the modern aqueous-ethanolic formulation of *Pelargonium sidoides* (EPs® 7630) shows substantial clinical efficacy against respiratory illnesses such as bronchitis and sinusitis through combined antibacterial and immunomodulatory action (Kolodziej, 2011). Because clinical-trial design is built around single-molecule drugs, herbal medicines that depend on the combinatorial effects of several phytochemicals are difficult to fit to that framework: researchers cannot easily isolate a single active molecule and must instead pursue more complex, unconventional regulatory pathways (Vaou et al., 2022).

Human safety is a further concern, compounded by the common misconception that herbal drugs are inherently safe, which encourages unmonitored use and an inadequate appreciation of adverse reactions, contraindications and herb-drug interactions (Ekor, 2014). Adverse effects are attributable not only to adulteration, heavy-metal contamination and misidentified plant species but also to the intrinsic toxicity of some plant species themselves (Balkrishna et al., 2024). The concept of drug safety is not new to Ayurveda, which has elaborate, textually scattered guidance on the consequences of herbal-medicine use - drug-drug interactions, overdosage and the like, including a dedicated category, *Abheshaja*, for substances deemed unsuitable as medicine (Ajanal et al., 2013). Even therapeutically useful secondary metabolites such as polyphenols can carry side effects: some polyphenols block iron uptake and inhibit digestive enzymes, and can interact with cytochrome P450 isoenzymes, reducing or inhibiting their activity and thereby directly affecting the metabolism of co-administered drugs (Duda-Chodak and Tarko, 2023).

### 8.2 Pharmacokinetic challenges and novel drug delivery systems

The effective concentrations reported in Sections 5 and 7 (for example, rutin hydrate at 128–1,024 μg/mL and quercetin at 125–1,000 μg/mL) are *in-vitro* exposure levels achieved by direct application to cultured bacteria, not demonstrated plasma or tissue concentrations in a living organism; whether they are reachable *in vivo* depends heavily on the bioavailability limits discussed below. Apart from bioavailability, instability and solubility of many phytochemicals are a barrier to clinical use. Their generally large size and polar nature make it difficult for them to cross endothelial linings, the gastrointestinal tract and the blood-brain barrier (Ahmad et al., 2021), and most are insoluble in water, so that oral administration often fails to deliver therapeutic concentrations to the target site once physiological barriers are accounted for; they are also degraded enzymatically and by gut microbes and metabolised rapidly, reducing their *in vivo* activity (Zuccari and Alfei, 2023). Nanocarriers such as liposomes and nanoparticles can overcome these limitations by encapsulating phytochemicals, increasing their stability and enabling targeted delivery (Parvin et al., 2025). Lipid nanoparticles, including solid lipid nanoparticles and nanostructured lipid carriers, encapsulate phytochemicals within a solid lipid matrix and cross biological membranes to enable targeted delivery, while liposomes, composed of phospholipids and cholesterol, can carry phytochemicals across biological membranes without compromising their activity (Ashfaq et al., 2023). Encapsulating or conjugating phytochemicals with such nanocarriers improves their gastrointestinal stability, absorption and dispersion, thereby enhancing their overall bioactivity (Ahmad et al., 2021).

### 8.3 Regulatory complexity for multicomponent botanicals

A further, largely structural barrier is regulatory. Drug approval frameworks worldwide are built around a single defined active pharmaceutical ingredient with a specified mechanism, purity profile and dose–response relationship; a polypharmacological botanical extract, by contrast, is an undefined mixture of many bioactive constituents whose relative proportions vary with source, season and processing (Vaou et al., 2022; Jarouche et al., 2019). This mismatch has practical consequences at every stage of development. Regulators typically require identification and quantification of “marker” compounds for quality control, yet no consensus exists on which of the many co-occurring metabolites in a plant extract should serve this role, or whether a single marker can meaningfully represent the activity of the whole mixture (Busia, 2024). Combination products consisting of two or more phytochemicals, or a phytochemical paired with a conventional antibiotic, face an even less-defined path, since most jurisdictions’ herbal-medicine and botanical-drug pathways were not designed with antimicrobial adjuvants in mind, and combination toxicology and interaction studies add further cost and complexity (Parveen et al., 2015). These regulatory obstacles compound, rather than substitute for, the reproducibility, safety and clinical-trial gaps discussed above, and they are a principal reason we characterise standardised-extract and adjuvant development, rather than novel single-molecule drug approval, as the more tractable near-term regulatory route for this chemistry.

## 9 Conclusion

Despite sustained action plans, global AMR has not meaningfully receded, and a structural cause lies in the single-target design of conventional antibiotics, which offers bacteria a single, well-defined route of escape. Ayurvedic pharmacology suggests a complementary logic. Its antibacterial plants, among them *C. procera*, *A. indica*, *O. sanctum*, *Acacia* species and *A. racemosus*, act through mixtures of secondary metabolites (flavonoids, tannins, saponins, phenolic acids and terpenoids) that engage several conserved bacterial targets at once: the cell wall, the membrane, essential enzymes, and notably the resistance machinery itself. Against such multi-target pressure, stepwise resistance is evolutionarily improbable.

The same multi-target chemistry addresses two failures of conventional therapy: the biofilm, which antibiotics penetrate poorly, and established resistance, which phytochemical adjuvants can partially reverse by degrading matrix, inhibiting efflux and suppressing β-lactamase. We therefore suggest that the most credible near-term role for Ayurvedic antibacterials is not as stand-alone replacements for antibiotics but as multi-target adjuvants and anti-biofilm agents that extend the useful life of the drugs we already possess. Realising that role will require confronting the field’s real limitations - reproducibility, contamination and the scarcity of clinical trials, with rigorous standardisation and properly designed clinical studies. Approached this way, the accumulated empirical knowledge of Ayurveda becomes not a rival to modern pharmacology but a source of chemistry uniquely suited to the multi-target problem that resistance has created.
